# The Fourteenth International Congress of Medicine

**Published:** 1901-12

**Authors:** 


### The Fourteenth International Congress of Medicine.

The Fourteenth International Congress of Medicine.—We have been asked to announce that in the above Congress, to be held at Madrid from the 23rd to the 30th of April, 1903, it has been decided to divide the eleventh of the sixteen sections into two independent sections; viz., 11a, Otology, and 11b, Rhino-laryngology. The sixteenth section (Médecine légale) will be entitled "Médecine légale et Toxicologie."

